# Comparative Analysis of LASIK Flap Diameter and its Centration Using Two Different Femtosecond Lasers

**Published:** 2019

**Authors:** Majid Moshirfar, Tanner W. Brown, Madeline B. Heiland, David B. Rosen, Yasmyne C. Ronquillo, Phillip C. Hoopes

**Affiliations:** 1 John A. Moran Eye Center, Department of Ophthalmology and Visual Sciences, School of Medicine, University of Utah Salt Lake City, UT, USA; 2 HDR Research Center, Hoopes Vision, Draper, UT, USA; 3 Utah Lions Eye Bank, Murray, UT, USA; 4 Health Science Center, School of Medicine, The University of Texas, Houston, TX, USA; 5 John Peter Smith Hospital, Fort Worth, TX, USA; 6 College of Medicine, The University of Arizona, Phoenix, AZ, USA

**Keywords:** Corneal Flap, Flap Morphology, Flap Diameter, Flap Centration, Flap Variance, Flap Predictability, Laser In Situ Keratomileusis, Laser, VisuMax Laser, Femtosecond Laser

## Abstract

The aim of this study was to compare the diameter, accuracy, variability, and centration with respect to the limbus of corneal flaps created by two femtosecond lasers, the VisuMax, and Wavelight FS200, for laser in situ keratomileusis (LASIK) and how these flaps affect visual outcomes. This is a retrospective chart review of flap morphology created during LASIK Surgery. Overall, 168 eyes underwent flap creation using the WaveLight FS200 laser, and on 189 eyes, the VisuMax laser was used. Of these total number, flap morphology was analyzed in a random sample of 158 eyes; 80 with the Visumax laser and 78 with the WaveLight FS200 laser. Intraoperative photos of the flaps taken by the Wavelight Allegretto EX500 were analyzed. Flap diameters and centration were measured using Adobe Acrobat Pro. All patients had visual acuity measurements including uncorrected distance visual acuity (UDVA), corrected distance visual acuity (CDVA), spherical equivalent refraction (SE) and refractive astigmatism recorded three months postoperatively. Greater than 90% of patients in both groups achieved a UDVA of 20/20 postoperatively. The mean difference between targeted and achieved flap diameter was 0.50 +/- 0.15 mm in the VisuMax group and 0.35 +/- 0.15 millimeters (mm) in the FS200 group (*P*<0.01). The flap diameters of the VisuMax group were more precise with a variance of 0.024 mm compared to a variance of 0.038 mm in the FS200 group (*P*<0.05). VisuMax flaps were more nasally displaced (log(N_A_/T_A_) = -0.21 +/- 0.10 mm) compared to the FS200 flaps (log(N_A_/T_A_) = 0.03 +/- 0.10 mm), (*P*< 0.01). We concluded that both the VisuMax and FS200 created flaps larger than the preoperative targeted diameter. VisuMax created corneal flaps that had a greater degree of deviation from the targeted diameter when compared to flaps from the FS200. However, there was less variance in the VisuMax flap diameter. In addition, VisuMax flaps were more nasally displaced. There were no statistically significant differences in visual outcomes when comparing the two femtosecond lasers.

## INTRODUCTION

Laser-assisted in situ keratomileusis (LASIK) is the most popular refractive surgery for the correction of myopia, hyperopia, and astigmatism and is one of the most common surgeries performed worldwide [1-3]. A critical step of LASIK is the creation of a corneal flap prior to stromal ablation with an excimer laser. Historically, this step was achieved with the blade of a mechanical microkeratome; however, this is no longer the standard technique, as the femtosecond laser, an ultra-short pulse laser, is superior in terms of flap production [4-9]. Flaps created by femtosecond lasers are more consistent and reproducible than the microkeratome and produce planar flaps (in contrast to the meniscus-shaped flaps seen with mechanical microkeratomes) [4, 6, 10, 11]. Multiple femtosecond lasers have been manufactured. While most studies compare flap profiles, thickness, and shape, this study examines the predictability and accuracy of targeted flap diameter, geometric centration with respect to the limbus, and visual outcomes for corneal flaps created by the Wavelight FS200 (Alcon, Fort Worth, Texas) and the VisuMax (Carl Zeiss Meditech AG, Jena, Germany). 

## METHODS

This study was a retrospective chart review of 160 eyes that had flaps created with the WaveLight FS200 laser (January 2016–April 2018) and 189 eyes that had flaps created with the VisuMax laser (January 2017–July 2017) for LASIK. Surgeries were performed at a single surgical site (Hoopes Vision) by two experienced surgeons (MM, PCH) in Draper, UT, USA. A random sample of 80 eyes for VisuMax and 78 eyes for FS200 was used for detailed morphological assessment of flap diameter and centration with respect to the limbus. 

Informed consent was obtained from all cases prior to surgery. The consent form included the release of their data for research and publication. All procedures adhered to the tenets of the Declaration of Helsinki. The Ethics Committee of HDR Research Center approved this study. Preoperative evaluation for all patients consisted of a full ophthalmic examination, which included: patient history, slit-lamp microscopy exam, dilated fundoscopy, ultrasound pachymetry, corneal topography/tomography, as well as measurement of uncorrected distance visual acuity (UDVA) and corrected distance visual acuity (CDVA). All patients were determined to be appropriate surgical candidates for LASIK.

LASIK was done using the standard technique. A corneal flap was created using either the VisuMax or FS200 femtosecond laser. After the flap was lifted, corneal ablation was performed using the Wavelight Allegretto EX500 (Alcon, Fort Worth, Texas) excimer laser with a 6.5 mm optical zone and blend zone to 9.0 mm. The eye was subsequently irrigated, and the corneal flap was repositioned. Flap creation with the FS200 laser was performed at 200 kiloHertz (kHz) in a raster pattern with bed energy of 0.7 Microjoule (μJ), and side cut energy of 0.7 μJ. Spot and line separations were 7.0 μm and 7.0 μm for the bed cut and 5.0 μm and 3.0 μm for the side cut. Hinge position of 90 degrees hinge angle of 50 degrees and hinge width of 3.8 mm were used. Targeted flap diameter was based on preoperative white to white distances measured using the OPD Scan III Wavefront Aberrometer (Marco, Jacksonville, Florida) and is as follows: patients with a white to white greater than 12.2 mm had a target diameter of 9.0 mm; white to white between 12.0 and 12.2 mm had a target diameter of 8.9 mm; white to white between 11.75 and 12.0 mm had a target diameter of 8.8 mm; and a white to white between 11.5 and 11.75 mm had a target diameter of 8.7 mm. Flap thickness of 100 μm was programmed with a 90-degree side cut angle.

**Figure 1:**
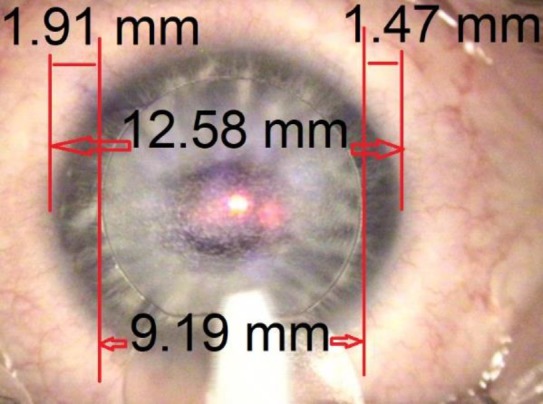
Measurements: White-to-White Distance (WWM), Flap Diameter (FM), Distance from Limbus to the Flap Margin on Nasal (NM) and Temporal (TM) Sides. Abbreviation: mm: millimeters

Flap creation with VisuMax was performed at 500 kHz in a spiral pattern with bed energy of 0.32 μJ, side cut energy 0.32 μJ, spot separation of 4.1 μm, track separation of 4.1 μm. Hinge position of 90 degrees, hinge angle of 58 degrees, and hinge width of 4.10 mm were used. Using the S cone with M settings, flap diameter of 8.1 mm and flap thickness of 110 μm was programmed with a 90-degree side cut angle. Postoperatively, patients were given Prednisolone acetate 1% (Falcon Pharmaceuticals Ltd., Fort Worth, TX) every hour for the first day and subsequently, four times a day for one week. Moxifloxacin 0.5% (Alcon Pharmaceuticals Ltd., Fort Worth, TX) was also given four times a day for one week. At a minimum, all patients were examined one day, one week, and one and three months postoperatively. Uncorrected distance visual acuity, CDVA, spherical equivalent refraction (SE), and refractive astigmatism were recorded at each visit. For flap morphology comparison, intraoperative photos of eyes were taken by the Wavelight Allegretto EX500 excimer laser post-flap creation with either the VisuMax or FS200. These photos were then converted to PDF files to be analyzed. Using Adobe (San Jose, CA, USA) Acrobat Pro DC, the intraoperative photos were magnified to 800%, and a grid was used with 2 mm distances between lines to aid in accurate measurements. Using the sliding measure caliper on the software set to a precision of 0.01 mm with line thickness reduced to 0.1 point (pt.), measurements were taken of white-to-white corneal diameter (WW_M_), flap diameter (F_M_), and distance from limbus to the flap margin on both nasal (N_M_) and temporal (T_M_) sides. A picture is included as an example (Fig. 1). The white- to- white corneal diameter measured on the intraoperative pictures were then compared to the white to white distances measured preoperatively using the OPD Scan III Wavefront Aberrometer, defined as white-to-white actual (WWA). Using these two measurements of corneal diameter, a scale ratio was created given the actual measurements of the other variables: actual flap diameter (F_A_), actual temporal distance (T_A_), and actual nasal distance (N_A_).

$F_{A}=(F_{M}\times {\mathrm{WW}}_{A})/{\mathrm{WW}}_{M}$

$T_{A}=(T_{M}\times {\mathrm{WW}}_{A})/{\mathrm{WW}}_{M}$

$N_{A}=(N_{M}\times {\mathrm{WW}}_{A})/{\mathrm{WW}}_{M}$

**Figure 2:**
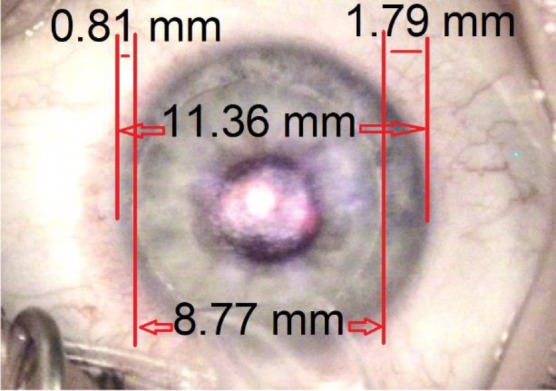
Example of Scaled Adobe Measurements of a Nasally Displaced Flap a Nasally Displaced Flap Created by the VisuMax (log(NM/TM) = log(1.15/2.54) <0. Abbreviation: mm: millimeters

Microsoft Excel was used for all calculations. To determine flap centration along the 180-degree meridian, the ratio of N_A_/T_A_ was used. The log of ratio (log(N_A_/T_A_)) was used to determine whether the flap was shifted towards the nasal side of limbus (log(N_A_/T_A_)< 0) or the temporal side of limbus (log(N_A_/T_A_)> 0) (Fig. 2). The Kolmogorov–Smirnov test was first used to check the normality of data. An independent two-sample t-test was used to analyze the difference between means, while a two-sample F test was used to analyze difference in variances. A *P*-value of <0.05 was used to determine significance for both statistical tests.

## RESULTS

**Visual Outcomes**

Nine standard graphs depicting visual outcome, safety, stability, achieved spherical equivalent, and astigmatic analysis of the patients who received LASIK flaps with the FS200 or the VisuMax are presented in Fig. 3A and 3B, respectively. Of the patients that returned for a 3-month postoperative visit, there were a total of 189 eyes that had a flap created with the VisuMax and 160 eyes that had a flap created with the FS200. The percentage of patients that achieved a postoperative UDVA of 20/20 or better was not significantly different between the Visumax (93%) and the FS200 (92%) (*P*>0.05). Three percent of VisuMax patients and 4% of FS200 patients lost one line of CDVA; again, there was no statistically significant difference (*P*>0.05). Additionally, there was no statistically significant difference between the proportion of patients that achieved a SE of within ± 0.50 D for the FS200 (91%) or the VisuMax (95%).

**Flap Diameter**

There were 41 patients and 80 eyes (40 right eyes [OD], 40 left eyes [OS]) that had flaps created with the VisuMax laser all with a target flap diameter of 8.1 mm. The mean ± standard deviation (SD) of diameter of these 80 flaps was 8.60 ± 0.15 mm (range 8.19 – 9.06). Overall, 43 patients and 78 eyes (39 OD, 39 OS) had flaps created with the FS200 laser. These eyes were subdivided into 4 groups depending on the target flap diameter that was pre-set based on the white-to-white distance. Three patients and 6 eyes (3 OD, 3 OS) had a target flap diameter of 8.7 mm. Ten patients and 20 eyes (9 OD, 11 OS) had a target flap diameter of 8.8 mm. Fifteen patients and 26 eyes (14 OD, 12 OS) had a target flap diameter of 8.9 mm. Sixteen patients and 26 eyes (13 OD, 13 OS) had a target flap diameter of 9.0 mm. Flap diameter means, SD, and range are reported in Table 1.

**Table 1:** Flap diameter Measurements for WaveLight FS200 Subgroups and VisuMax

Target Flap Diameter (mm)	N	Mean ± SD (mm)	Variance	Range (mm)	Mean ± SD (mm)
FS200					
8.7	6	9.18 ± 0.13	**0.018**	9.02 – 9.36	0.48 ±0.13
8.8	20	9.13 ± 0.20	**0.042**	8.66 – 9.42	0.35 ± 0.17
8.9	26	9.24 ± 0.20	**0.038**	8.69 – 9.46	0.37 ± 0.14
9.0	26	9.28 ± 0.18	**0.034**	8.62 – 9.59	0.31 ± 0.13
All	78	9.22 ± 0.20	**0.038**	8.62 – 9.59	0.35 ± 0.15
Visumax					
8.1	80	8.60 ± 0.15	**0.024**	8.19 – 9.06	0.50 ± 0.15

Abbreviations: mm: millimeters; SD: standard deviation; P-values <0.05 are in bold. N: Number of eyes

**Flap Diameter Predictability**

The absolute values of the differences between actual and targeted flap diameters for each laser are compared in Table 1. The mean difference from target flap diameter for the FS200 (0.35 ± 0.15 mm) was less than the VisuMax (0.50 ± 0.15 mm), (*P*<0.01). The median flap diameter, first and third quartiles, minimum, and maximum values for each targeted diameter are shown in Fig. 4. 

**Figure 3A:**
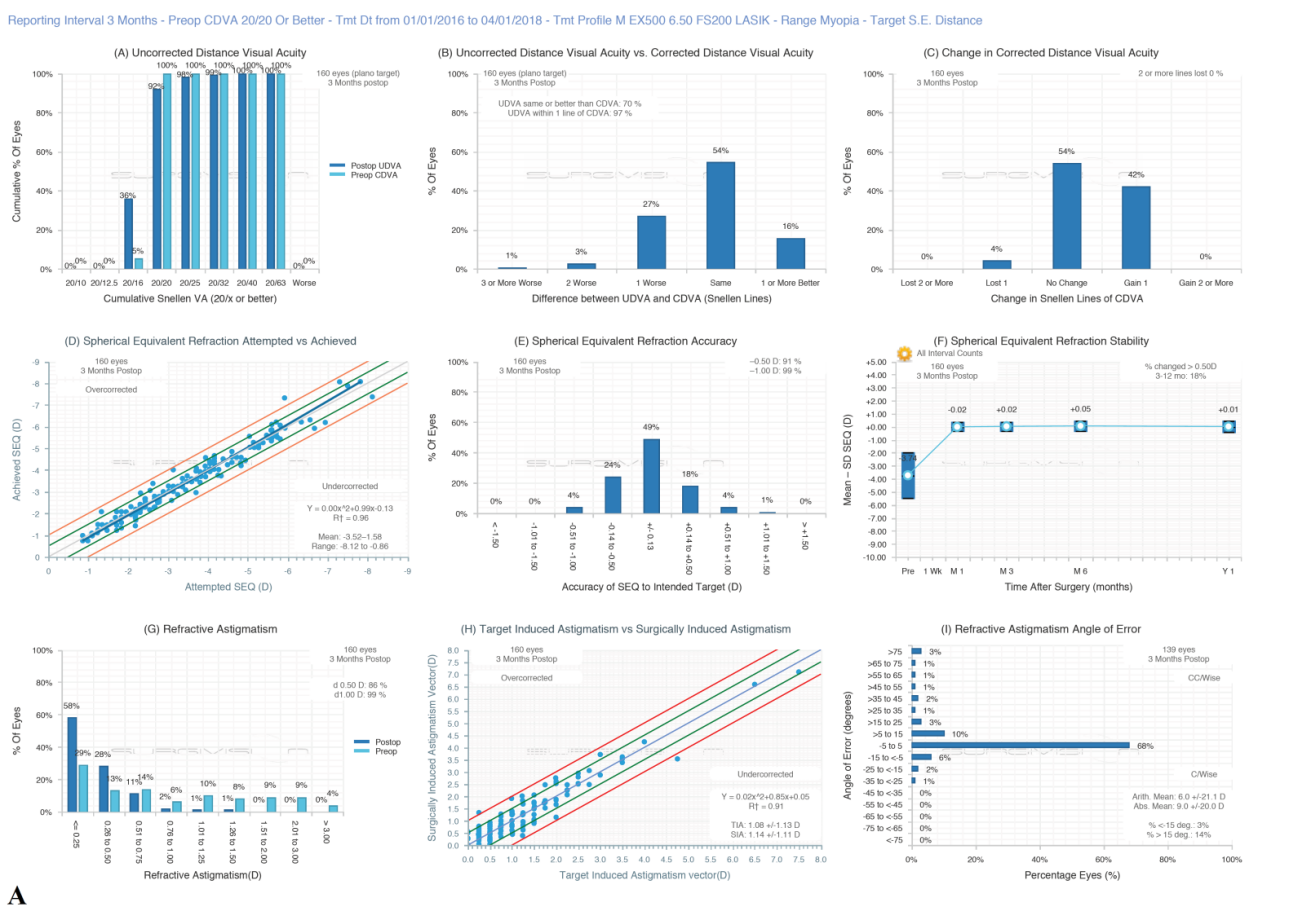
Nine standard graphs reporting refractive surgery outcomes of 168 eyes treated by the WaveLight FS200 laser. Visual outcomes (uncorrected distance visual acuity= UDVA, UDVA versus corrected distance visual acuity= CDVA, Change in CDVA), spherical equivalent refraction (SER), SER Attempted versus Achieved, SER accuracy, SER stability, refractive astigmatism, and surgically induced astigmatism (SIA) are depicted

**Figure 3B:**
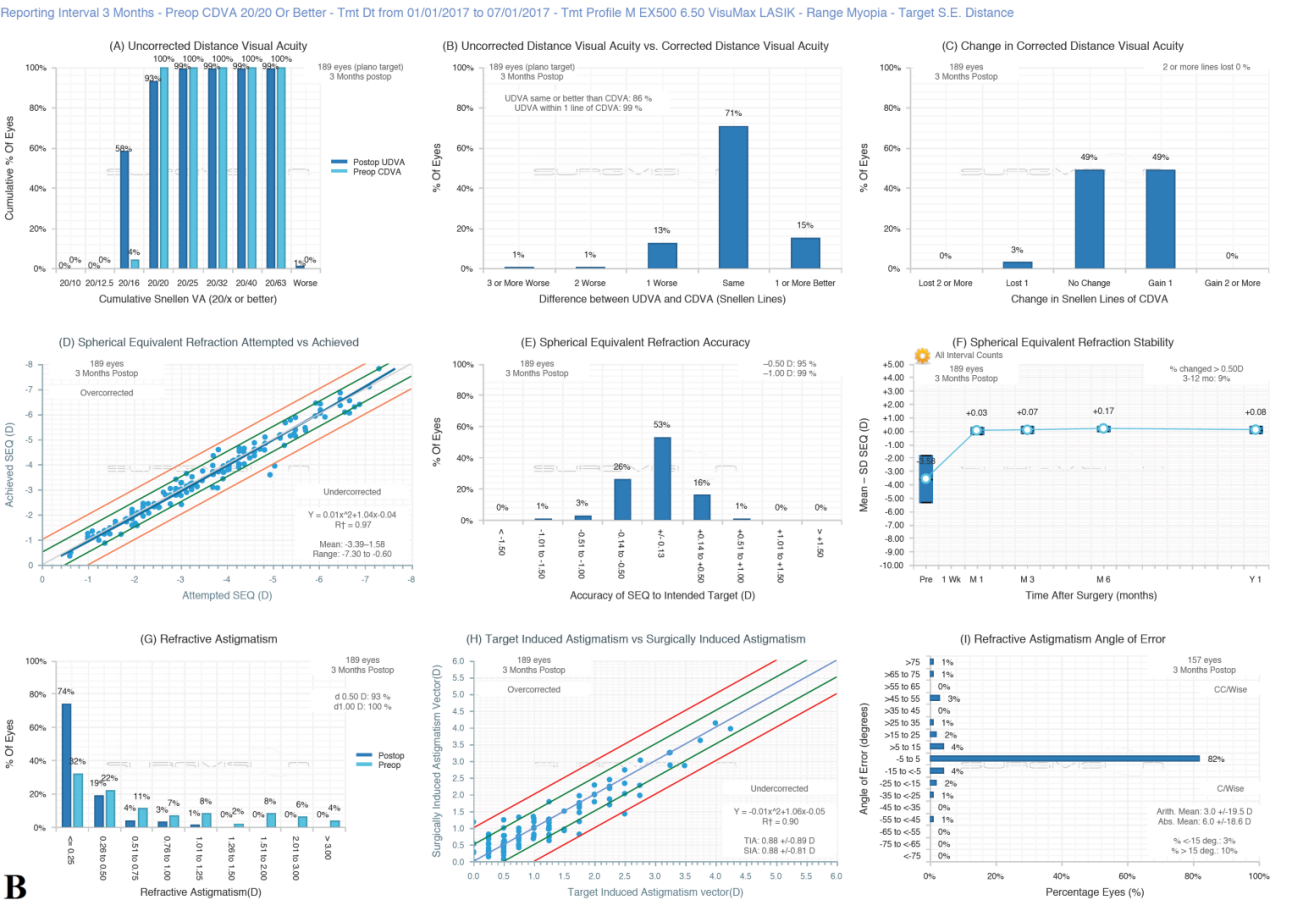
Nine standard graphs for reporting refractive surgery outomes of 189 eyes treated by the VisuMax laser. Visual outcomes (uncorrected distance visual acuity= UDVA, UDVA versus corrected distance visual acuity= CDVA, Change in CDVA), spherical equivalent refraction (SER), SER Attempted versus Achieved, SER accuracy, SER stability, refractive astigmatism, and surgically induced astigmatism (SIA) are depicted

**Figure 4:**
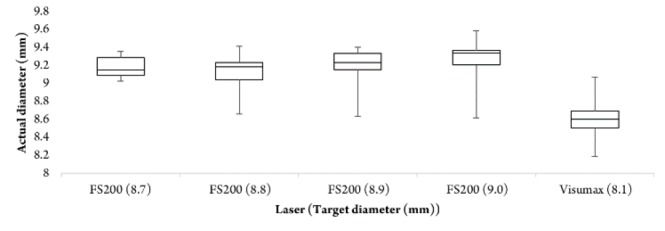
Comparison of targeted flap diameter and achieved flap diameter for the WaveLight FS200 and VisuMax

**Flap Diameter Variability**

**Figure 5:**
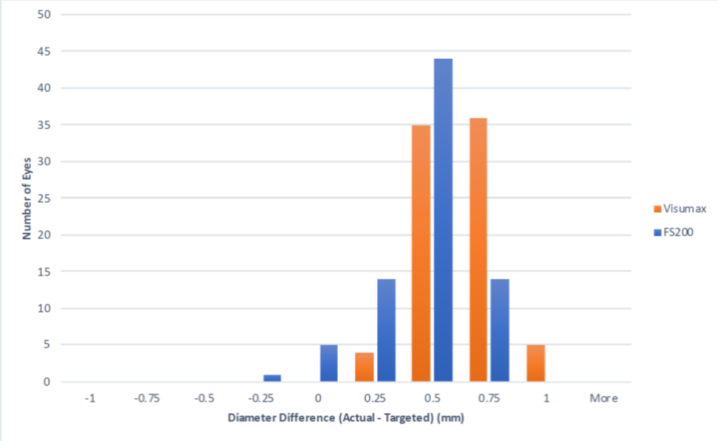
Analysis of variance in targeted flap diameter and achieved flap diameter for the WaveLight FS200 and VisuMax

Although the VisuMax flaps were less predictable in targeted diameter (*P*<0.01) when analyzing the variance of the differences between targeted and achieved diameter it was found that the VisuMax flap measurements showed less variance (0.024) than the collective FS200 group (0.038), (*P*<0.05). This variance is shown in Fig. 5. 

When the FS200 flaps were divided into subgroups, the only statistically significant difference in variance was between the VisuMax group (0.024) and the FS200 group pre-set to 8.8 mm (0.042) (*P*<0.05). All other FS200 groups had variances from the VisuMax group that was not statistically different from one another (Table 2).

**Table 2:** p-values of Variance Comparison between WaveLight FS200 Subgroups and VisuMax

Groups	8.7 mm	8.8 mm	8.9 mm	9.0 mm
(FS200) 8.8 mm	P = 0.18	X	X	X
(FS200) 8.9 mm	P = 0.20	P = 0.42	X	X
(FS200) 9.0 mm	P = 0.25	P = 0.31	P = 0.37	X
8.1mm (Visumax)	P = 0.41	**P = 0.04**	P = 0.06	P = 0.12

Note: X denotes that the P-value for that combination has already been stated in the table. P-values were not calculated for two of the same values in mm. Abbreviations: mm: millimeters; P-values <0.05 are in bold.

**Flap Centration**

**Figure 6:**
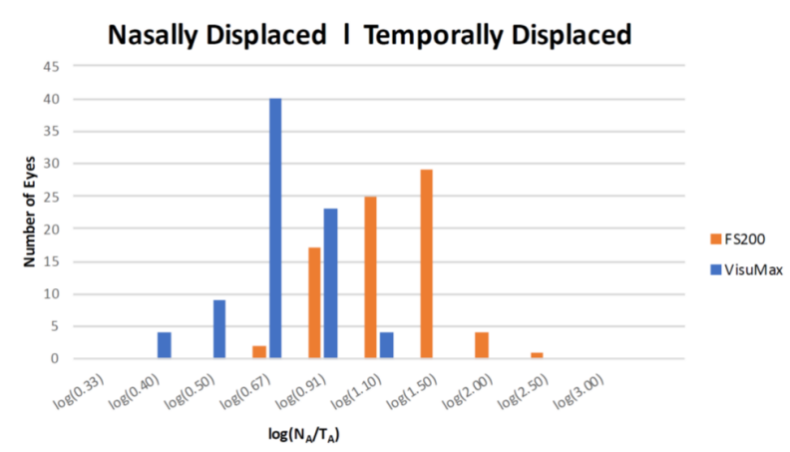
Depiction of flap centration (nasal or temporal displacement) for the WaveLight FS200 and VisuMax in respect to the limbus

**Figure 7:**
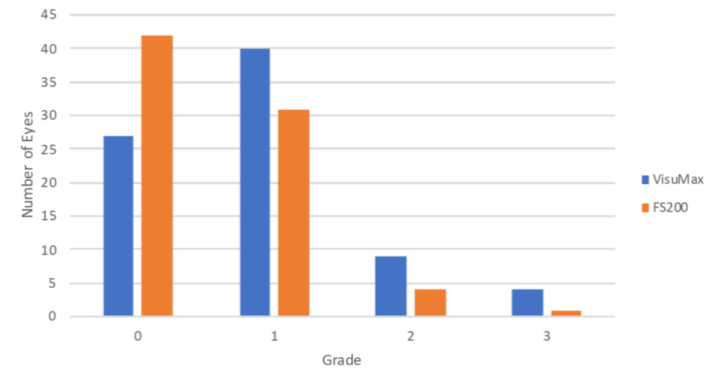
**Severity of flap displacement from the limbus for the WaveLight FS200 and VisMax. grade 0: log (N**_A_ **/ T**_A_ **) from -0.04 to 0.04, grade 1: log(N**_A_ **/ T**_A_ **) from -0.18 to 0.18, grade 2: log(N**_A_ **/ T**_A_ **) from -0.30 to 0.30, grade 3: log(N**_A_ **/ T**_A_ **) from -0.40 to 0.40**

The mean flap centration with respect to the limbus at the 180-degree meridian for each group was calculated by averaging N_A_/T_A_ for every eye and then converting to log(N_A_/T_A_). Flaps created with the VisuMax (-0.21 ± 0.10 mm) were more nasally displaced than the flaps created by the FS200 (0.03 ± 0.10 mm), (*P*<0.01). This degree of displacement is shown in Fig. 6. In order to characterize the degree of flap displacement, a sliding scale was created where grades 0, 1, 2, and 3 correspond to increasing displacement of the flap from the geometric center of the limbus. The grades are defined as follows: grade 0: log (N_A_/ T_A_) from -0.04 to 0.04, grade 1: log(N_A_/ T_A_) from -0.18 to 0.18, grade 2: log(N_A_/ T_A_) from -0.30 to 0.30, grade 3: log(N_A_/ T_A_) from -0.40 to 0.40. The flaps created by the VisuMax laser showed a higher degree of displacement from the geometric center of the limbus. The grades of displacement are shown in Fig. 7.

**Figure 8:**
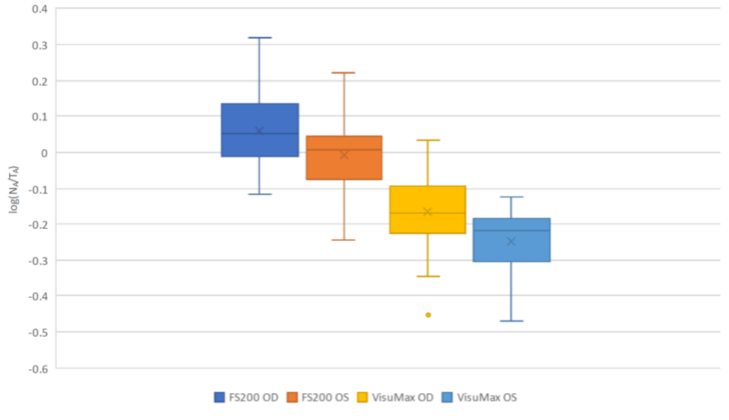
Variability of flap displacement in left (OS) and right eyes (OD) for WaveLight FS200 and VisuMax

Differences in flap displacement depending on laterality of the eye were noted. Using the mean values for log (N_A_/ T_A_) in the FS200 laser group, right eyes (log (N_A_/T_A_) =0.06) were more temporally displaced than the left eyes (log (N_A_/ T_A_) = -0.01). In the VisuMax group, both left and right eyes were nasally displaced; however, the left eyes (log (N_A_/ T_A_) = -0.25) were more displaced than the right eyes (log (N_A_/ T_A_) = -0.17) (Fig. 8).

## DISCUSSION

The primary objective of the study was to determine how flaps created by the VisuMax and WaveLight FS200 femtosecond lasers differed in terms of achieved diameter and centration with respect to the geometric center of the limbus. The results of the study showed that both lasers created a larger flap diameter than was targeted preoperatively, but the VisuMax created a flap that was larger in achieved diameter compared to the FS200. This can be attributed to the concavity of the curved applanation cone used with VisuMax platform. However, the VisuMax created flaps with less variance (0.024) in diameter across all measured eyes. Any deviation from target diameter and target position of the flap are relevant because an optimal corneal flap during LASIK is essential to provide satisfactory refractive outcomes while minimizing potential complications [12]. Unlike mechanical microkeratomes, femtosecond lasers create corneal flaps without the use of a blade. Instead, they focus ultra-short impulses of light energy on a fixed depth of the cornea, which causes photodisruption while sparing surrounding tissue [9]. Numerous articles have been published on flap profiles and compared to the mechanical microkeratome, femtosecond lasers create thinner flaps with greater consistency and predictability [4, 6, 8, 13, 14]. Consistent and thinner flaps result in increased speed of visual recovery after surgery [4, 15]. 

Flaps created by different femtosecond lasers have been compared using methods including optical coherence tomography and ultrasound pachymetry. A study comparing the thickness of corneal flaps by the VisuMax and FS200 lasers found that although both created flaps that were consistent, the FS200 flaps were significantly thinner than those of the VisuMax [16]. However, differences in flap thickness are not unique to the VisuMax and FS200. Similar findings in flap thickness differences between two femtosecond lasers, the iFS 150-kHz and LenSx, demonstrated the iFS 150-kHz laser to make consistently thinner flaps [17]. 

A unique aspect of this study was the comparison of flap symmetry with respect to the limbus. In our study, the VisuMax laser created flaps that were significantly more nasally displaced than the FS200 laser. A possible reason for nasal displacement is the curved corneal interface and comparatively gentle suction of the VisuMax. This interface allows patients to maintain fixation during flap creation, centering the flap on the visual axis as opposed to the pupil center. The visual axis and the pupil center are commonly different, and the degree to which they are different is defined by the angle kappa [18]. Although the flaps created by the VisuMax laser appear displaced relative to the pupil center, they may be more aligned with the visual axis. This alignment becomes important because proper centration of laser ablation during LASIK surgery is necessary to achieve good refractive outcomes and reduce post-operative adverse effects [19]. 

A potential risk involved in a displaced flap is damage to the limbal vessels (especially important in patients that have corneal pannus). If this occurs, it can result in intraoperative bleeding, which can prolong the surgery, lead to complications during ablation, and rarely, cause diffuse lamellar keratitis [20]. Although no patients included in the study suffered from intraoperative bleeding, the presence of corneal pannus should be a consideration when determining the placement of a corneal flap. 

A significant concern with larger than intended flap diameters is the possible structural instability within the cornea and an increase in dry eye. The cornea is densely populated with nerve fibers that moderate tear production and wound healing [21, 22]. Because nerve damage leads to symptoms of dry eye, and a larger flap diameter could lead to increased nerve damage, it is reasonable that a larger flap diameter could lead to increased risk of post-operative dry eye. Based on our findings, authors recommend that perhaps we should target dimensions of flaps smaller than what are currently used in clinical settings for these two femtosecond laser platforms. However, in this retrospective study, level of ocular surface dryness was not evaluated.

While the goal of the study was to compare the accuracy of flap diameter created by two different lasers, it is crucial to understand whether or not flap diameter, regardless of the laser, impacts visual outcomes post-LASIK. Despite the variances in flap morphology when comparing the FS200 and the VisuMax lasers, there were no statistically significant differences in UDVA, CDVA, SE, and refractive astigmatism three months postoperatively. These findings suggest that the differences in flap morphology do not directly translate to differences in visual outcomes. However, there was a lack of analysis of higher-order aberrations (HOAs) and contrast sensitivity in this study in relation to flap diameter or placement. One study that aimed to answer this question found that there was no difference in postoperative refractive errors, visual acuity, or root mean square of total HOAs (HO-RMS) whether an 8.1 mm or 8.6 mm flap diameter was used [23]. Note however that certain HOAs such as spherical aberration (Z12) and vertical coma (Z7) were increased regardless of the flap diameter used. 

Limitations of this study include the absence of data on flap morphology characteristics such as flap thickness. Comparisons of flap morphology typically include assessing thickness and uniformity of flaps; however, this was beyond the scope of this study. A potential source of systematic error in the data was the use of intraoperative pictures and the measurement tool to record WW_M_, F_M_, N_M_, and T_M_. It is possible that shape distortion existed in the pictures taken by the EX500 laser, have led to inaccurate measurements of flap diameter. Additionally, the actual distances (F_A_, N_A_, and T_A_) were based on the scale ratio of the WWA, measured by the OPD Scan III Wavefront Aberrometer. Inaccuracies of this measurement would have led to systematic inaccuracies in all measurements. Although this would have affected the accuracy of flap diameter measurements, it is likely that the distortions across all pictures were constant and therefore the results are still valid. While the geometric symmetry analysis would not be affected, variation in surgeon technique and dominant-handedness may be another confounding factor in flap centration variance. Centration along the vertical 90-degree meridian to determine displacement superiorly or inferiorly was not evaluated. Since the flaps were already retracted, it was difficult to view the superior position of the hinge with respect to the vertical meridian retrospectively from the existing photographs. This is was a limitation due to the retrospective nature of this study. Although the sample size was relatively large, there was a disadvantage in the VisuMax arm of the study. For the FS200, we had four diameter options based on the white-to-white; however, the VisuMax did not have subgroups. For the VisuMax only the S cone with the M setting was used, and larger flap diameter using the M cone with the L setting was never investigated. This is another limitation in terms of the comprehensiveness of this project. Perhaps a prospective study accounting for these variables would increase the power of the study.

## CONCLUSION

The authors do not try to advocate for perfect symmetry when it comes to flap creation. A nasally oriented flap could be beneficial if future data suggests that ablation with respect to the visual axis results in better patient outcomes than the pupillary axis. This report focuses on the different patterns of flaps observed with the use of different femtosecond lasers. There are opportunities for more prospective research in flap alignment relative to the visual versus pupillary axis.

## DISCLOSURE

Ethical issues have been completely observed by the authors. All named authors meet the International Committee of Medical Journal Editors (ICMJE) criteria for authorship of this manuscript, take responsibility for the integrity of the work as a whole, and have given final approval for the version to be published. No conflict of interest has been presented.

## Funding/Support:

This research has been supported by Research to Prevent Blindness (New York, USA).

## References

[1] Sugar A, Rapuano CJ, Culbertson WW, Huang D, Varley GA, Agapitos PJ (2002). Laser in situ keratomileusis for myopia and astigmatism: safety and efficacy: a report by the American Academy of Ophthalmology. Ophthalmology.

[2] Varley GA, Huang D, Rapuano CJ, Schallhorn S, Boxer Wachler BS, Sugar A (2004). LASIK for hyperopia, hyperopic astigmatism, and mixed astigmatism: a report by the American Academy of Ophthalmology. Ophthalmology.

[3] Solomon KD, Fernandez de Castro LE, Sandoval HP, Biber JM, Groat B, Neff KD (2009). LASIK world literature review: quality of life and patient satisfaction. Ophthalmology.

[4] Pajic B, Vastardis I, Pajic-Eggspuehler B, Gatzioufas Z, Hafezi F (2014). Femtosecond laser versus mechanical microkeratome-assisted flap creation for LASIK: a prospective, randomized, paired-eye study. Clin Ophthalmol.

[5] McAlinden C (2012). Corneal refractive surgery: past to present. Clin Exp Optom.

[6] Kezirian GM, Stonecipher KG (2004). Comparison of the IntraLase femtosecond laser and mechanical keratomes for laser in situ keratomileusis. J Cataract Refract Surg.

[7] Aristeidou A, Taniguchi EV, Tsatsos M, Muller R, McAlinden C, Pineda R (2015). The evolution of corneal and refractive surgery with the femtosecond laser. Eye Vis (Lond).

[8] Zhang Y, Chen YG, Xia YJ (2013). Comparison of corneal flap morphology using AS-OCT in LASIK with the WaveLight FS200 femtosecond laser versus a mechanical microkeratome. J Refract Surg.

[9] Torrisi L, Roszkowska AM, Urso M, Signorino A, Aragona P, Cutroneo M (2018). Use of the Femtosecond Lasers in Ophthalmology. EPJ Web of Conferences.

[10] Huhtala A, Pietila J, Makinen P, Uusitalo H (2016). Femtosecond lasers for laser in situ keratomileusis: a systematic review and meta-analysis. Clin Ophthalmol.

[11] Salomao MQ, Wilson SE (2010). Femtosecond laser in laser in situ keratomileusis. J Cataract Refract Surg.

[12] Melki SA, Azar DT (2001). LASIK complications: etiology, management, and prevention. Surv Ophthalmol.

[13] Kanellopoulos AJ, Asimellis G (2013). Three-dimensional LASIK flap thickness variability: topographic central, paracentral and peripheral assessment, in flaps created by a mechanical microkeratome (M2) and two different femtosecond lasers (FS60 and FS200). Clin Ophthalmol.

[14] Colombo-Barboza MN, Colombo-Barboza GN, Colombo-Barboza LR, Matuoka ML, Neto AL, de Freitas D (2018). Reproducibility of laser in situ keratomileusis flap thickness using a new multifunctional femtosecond laser platform and correlation with clinical preoperative measurements. J Cataract Refract Surg.

[15] Eleftheriadis H, Prandi B, Diaz-Rato A, Morcillo M, Sabater JB (2005). The effect of flap thickness on the visual and refractive outcome of myopic laser in situ keratomileusis. Eye (Lond).

[16] Zheng Y, Zhou Y, Zhang J, Liu Q, Zhai C, Wang Y (2015). Comparison of laser in situ keratomileusis flaps created by 2 femtosecond lasers. Cornea.

[17] Parafita-Fernandez A, Garcia-Gonzalez M, Katsanos A, Gros-Otero J, Teus M (2019). Two Femtosecond Laser LASIK Platforms: Comparison of Evolution of Visual Acuity, Flap Thickness, and Stromal Optical Density. Cornea.

[18] Moshirfar M, Hoggan RN, Muthappan V (2013). Angle Kappa and its importance in refractive surgery. Oman J Ophthalmol.

[19] Tsai YY, Lin JM (2000). Ablation centration after active eye-tracker-assisted photorefractive keratectomy and laser in situ keratomileusis. J Cataract Refract Surg.

[20] Azar D, Koch D (2003). LASIK : Fundamentals, Surgical Techniques, and Complications.

[21] Lee BH, McLaren JW, Erie JC, Hodge DO, Bourne WM (2002). Reinnervation in the cornea after LASIK. Invest Ophthalmol Vis Sci.

[22] Denoyer A, Landman E, Trinh L, Faure JF, Auclin F, Baudouin C (2015). Dry eye disease after refractive surgery: comparative outcomes of small incision lenticule extraction versus LASIK. Ophthalmology.

[23] Zhang YL, Liu L, Cui CX, Hu M, Li ZN, Cao LJ (2013). Comparative study of visual acuity and aberrations after intralase femtosecond LASIK: small corneal flap versus big corneal flap. Int J Ophthalmol.

